# The effect of prehabilitation on the postoperative outcomes of patients undergoing colorectal surgery: A systematic review and meta-analysis

**DOI:** 10.3389/fonc.2022.958261

**Published:** 2022-07-29

**Authors:** Xiaoting Zhang, Shaokang Wang, Wentao Ji, Huixian Wang, Keqian Zhou, Zhichao Jin, Lulong Bo

**Affiliations:** ^1^ Faculty of Anesthesiology, Changhai Hospital, Naval Medical University, Shanghai, China; ^2^ Department of Emergency, Changhai Hospital, Naval Medical University, Shanghai, China; ^3^ College of Basic Medicine, Naval Medical University, Shanghai, China; ^4^ Department of Health Statistics, Naval Medical University, Shanghai, China

**Keywords:** colorectal surgery, complications, functional capacity, meta-analysis, prehabilitation, systematic review

## Abstract

**Study objective:**

Prehabilitation is analogous to marathon training and includes preoperative preparation for exercise, as well as nutrition and psychology. However, evidence-based recommendations to guide prehabilitation before colorectal surgery are limited. We aimed to evaluate the effect of prehabilitation on the postoperative outcomes of patients undergoing colorectal surgery.

**Design:**

This study is a systematic review and meta-analysis.

**Methods:**

The PubMed, Embase, and Cochrane databases were searched for studies reporting the effect of prehabilitation strategies versus standard care or rehabilitation in patients undergoing colorectal surgery. The primary outcomes were overall postoperative complications and length of hospital stay (LOS), and the secondary outcome was functional capacity (measured using the 6-min walk test [6MWT]) at 4 and 8 weeks after surgery.

**Main results:**

Fifteen studies with 1,306 participants were included in this meta-analysis. The results showed no significant reduction in the number of overall postoperative complications (risk ratio = 1.02; 95% confidence interval [CI] = 0.79–1.31; *p* = 0.878) or LOS (standardized mean difference = 0.04; 95% CI = −0.11 to 0.20; *p* = 0.589) in patients who underwent colorectal surgery with or without prehabilitation strategy. Additionally, there were no significant differences in the functional capacity estimated using the 6MWT at 4 and 8 weeks postoperatively.

**Conclusions:**

Prehabilitation did not significantly affect the number of postoperative complications, LOS, or functional capacity of patients undergoing colorectal surgery. Whether prehabilitation should be recommended deserves further consideration.

**Systematic Review Registration:**

https://www.crd.york.ac.uk/PROSPERO/display_record.php?RecordID=290108, identifier CRD42021290108

## Introduction

Colorectal cancer (CRC) ranks third in terms of global cancer incidence and is the second leading cause of cancer-related mortality according to Global Cancer Statistics 2020. More than 1.9 million CRC cases were diagnosed and over 900,000 CRC-related deaths occurred in 2020 (1). Surgery is the primary curative treatment for CRC. However, adverse outcomes following colorectal surgery are common and costly despite advances in surgical techniques, perioperative care, enhanced recovery after surgery (ERAS) protocols, and rehabilitation strategies (2).

Prehabilitation was recently proposed to optimize preoperative conditions, thereby improving postoperative outcomes. Unlike ERAS and rehabilitation, which mainly focus on the postoperative period, prehabilitation can help patients enhance their physiological reserves and improve their functional capacity before surgery (3, 4) using interventions focusing on nutrition, exercise, and psychosocial components. Thus, prehabilitation can be thought of as training before a marathon owing to the multidimensional aspects of preoperative preparation, which may enable patients to optimize their surgical eligibility and improve their surgical outcomes (5).

Several previous studies have reported the potential advantages of prehabilitation for various surgical procedures (6, 7). However, the number of meta-analyses on the prehabilitation of patients undergoing colorectal surgery is currently limited (8–10). These studies also reported conflicting results regarding the relationship between prehabilitation and length of hospital stay (LOS). Thus, whether prehabilitation strategies are beneficial and which detailed type of prehabilitation strategies can affect the outcomes of patients undergoing colorectal surgery positively remain unknown. Therefore, generating and evaluating the best evidence for prehabilitation strategies concerning colorectal surgery is imperative.

This systematic review and meta-analysis aimed to determine the effect of prehabilitation on the postoperative outcomes of patients undergoing colorectal surgery. Our findings may support evidence-based medical practices and guide clinicians’ decisions.

## Methods

### Study design

This systematic review and meta-analysis was conducted in accordance with the Preferred Reporting Items for Systematic Reviews and Meta-Analyses (PRISMA) guidelines and registered in the International Prospective Register of Systematic Reviews (CRD42021290108) (11).

### Literature search

A systematic literature search of PubMed, Embase, and Cochrane databases for papers published from inception to 25 January 2022, was performed without language limitation. We sought to include studies exploring prehabilitation strategies in patients undergoing colorectal surgery. The search was constructed using the PICO (patient, intervention, comparison, and outcome) framework: patient (adults undergoing colorectal surgery), intervention (prehabilitation strategies), comparator (standard care or rehabilitation only), and outcome (primary: overall complication rates and LOS). The full literature search strategy is presented in **Table S1**. A database of privately and publicly funded clinical studies conducted worldwide was also sought by screening trial registries (https://www.clinicaltrials.gov/ and https://trialsearch.who.int/). Manual backward searches of references from the primary studies and other relevant systematic reviews were also conducted. After the database search and sourcing of the manuscripts were complete, all original publications were downloaded into a single reference list, and duplicates were removed.

### Study selection criteria

Studies that allocated adult participants (aged ≥18 years) undergoing colorectal surgery to receive prehabilitation strategies versus standard care or rehabilitation were eligible for inclusion in this study. The inclusion criteria were as follows: (1) studies involving patients undergoing colorectal surgery; (2) prehabilitation intervention included exercise, nutritional optimization, or psychological support alone or in combination as defined by original studies; (3) control groups included standard care, placebo, or postoperative rehabilitation only; and (4) randomized controlled trials (RCTs) and quasi-RCTs, such as those that allocate participants to groups based on the location of residence or date of assessment. The exclusion criteria were as follows: (1) no available full-text article, (2) reviews or protocol manuscript, (3) secondary analysis, (4) no defined outcomes, and (5) duplicate records.

### Data extraction

The data extraction form was piloted by all reviewers and revised by consensus. Two authors (XZ and SW) independently and parallelly screened all titles and abstracts. Articles were considered for full-text review if they met the study inclusion criteria or could not be excluded based on the abstract alone. Discrepancies were addressed by a discussion with a third reviewer (LB) to reach a consensus.

The data extraction form gathered the following information: author’s name, country, publication year, type of study design, study aim and design, participants’ data, details of prehabilitation intervention and comparison groups, overall complications, LOS, and 6-minute walk test (6MWT) at 4 and 8 weeks.

### Assessment of methodological quality and risk of bias

Two authors independently assessed the quality of the included articles using the Cochrane Collaboration tool for risk of bias assessment. Each study was rated as unclear, low risk, or high risk for random sequence generation, allocation concealment, blinding, attrition, and selective outcome reporting. In cases of disagreement, a consensus was reached through discussion.

Publication bias was visually assessed using funnel plots and quantitatively calculated using the Egger’s, Begg’s, and Harbord’s tests (12). The certainty of the evidence for outcomes was examined using the grading of recommendations assessment, development, and evaluation approach (13).

### Primary and secondary outcomes

The primary outcomes were overall postoperative complications and LOS. Postoperative complications (e.g., pneumonia, urinary tract infection, and hemorrhage) following colorectal surgery and postoperative LOS, which was calculated from the date of surgery until hospital discharge, were assessed.

The secondary outcome was functional capacity assessed using 6MWT performed 4 and 8 weeks postoperatively. Patients were instructed to walk back and forth at a certain length of the hallway for 6 min at a pace that would tire them by the end of the walk. The distance in meters reflects the physical function of the patients (14).

### Statistical analysis and data management

Outcome data were pooled using the Mantel–Haenszel method based on a random- or fixed-effects model when available from at least two trials. Heterogeneity between studies was quantified using the *I* ^2^ statistic. Random-effects models were prioritized if *I* ^2^ > 40% or *p* < 0.10 for significant heterogeneity. Statistical significance was set at two-sided *p* < 0.05.

Forest and funnel plots were generated using Stata 13.1 (StataCorp LLC, College Station, Texas, United States) and RevMan 5.3 software (The Nordic Cochrane Center, The Cochrane Collaboration, Copenhagen, Denmark). The principal summary measures were risk ratios and standard mean differences (SMDs) with 95% confidence intervals (CIs) for dichotomous and continuous variables, respectively. Where means and standard deviations could not be extracted from the included trials, they were estimated from medians and interquartile ranges using methods described by Wan and others (15). Funnel plots were constructed to detect publication biases. There was no significant publication bias if the two sides were symmetrical; otherwise, a publication bias was possible.

For the primary outcomes, sensitivity analyses were performed using Stata 13.1 with a “leave-one-out” approach, in which all studies were iteratively removed one at a time to analyze their influence on both pooled estimates and heterogeneity. For the overall complication rate, the source of heterogeneity was further explored with a meta-regression, and the possible covariants (year of publication, age, type of control, or geographical location) were tested. Subgroup analyses were also conducted based on the exact type of control and intervention strategies to identify potential influencing factors.

Trial sequential analysis (TSA) was performed for both dichotomous and continuous primary outcomes to reduce the possible risks of random errors owing to insufficient sample size and repeated significance testing of pooled data. TSA software version 0.9.5.10 beta (Copenhagen Trial Unit, Centre for Clinical Intervention Research, Copenhagen, Denmark) was used to perform the analysis and estimate the required information size (RIS) for this meta-analysis. Monitoring boundaries were used to determine whether the *p*-values in the meta-analyses sufficiently demonstrated the anticipated effect.

## Results

### Study selection

**Figure 1:**
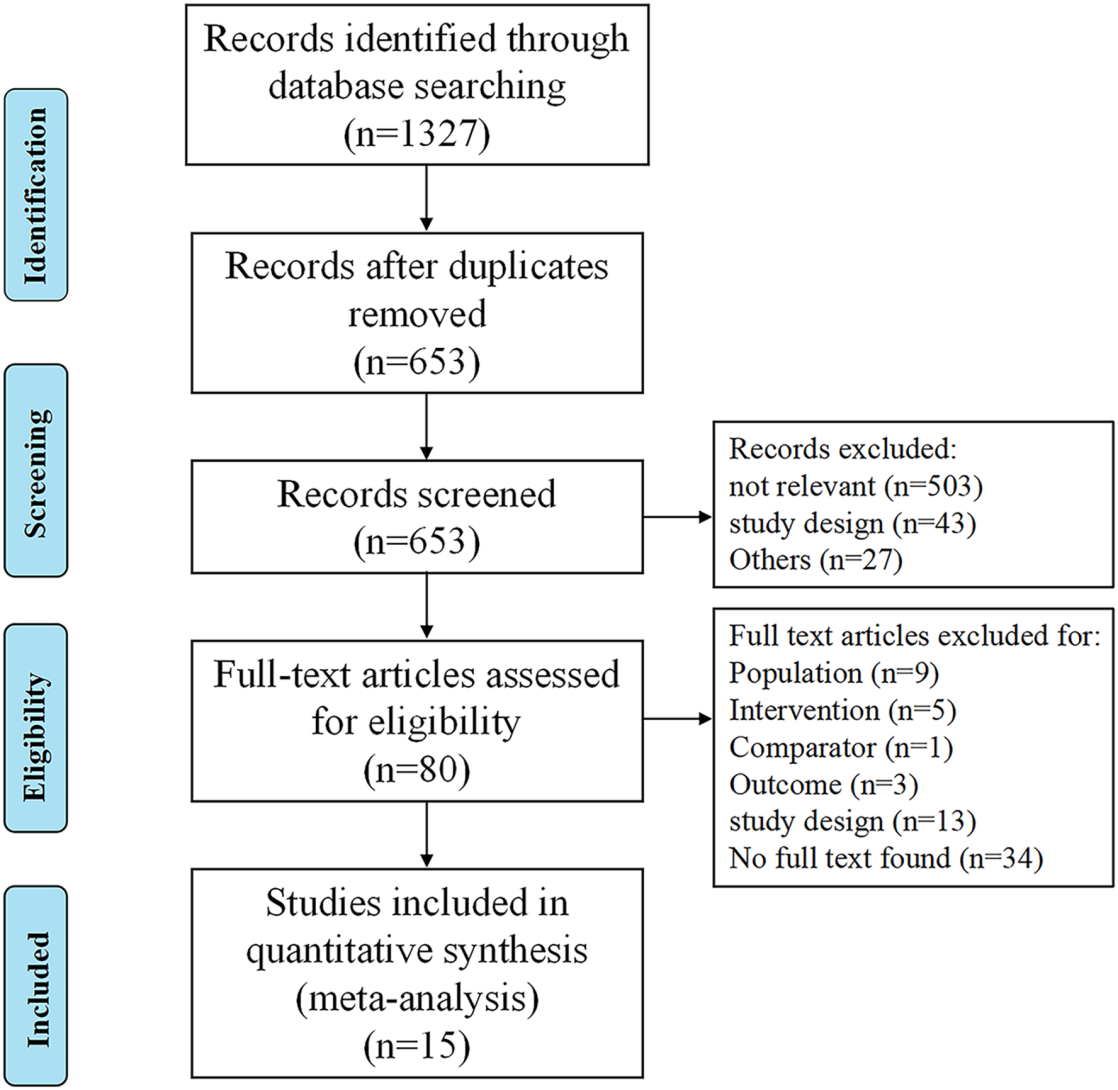
PRISMA flow diagram of the selection and inclusion process.

The literature search identified 653 non-duplicate citations (**Figure 1**), of which 573 were excluded after the abstract screening. Thus, 80 full-text articles were retrieved and assessed for eligibility, of which 65 were excluded because of the ineligible study population (*n* = 9), no utilization of prehabilitation strategy (*n* = 5), ineligible comparator (*n* = 1), lack of outcome assessment (*n* = 3), incorrect study design (*n* = 13), or unavailability of the full text (*n* = 34). In total, 15 trials were included in the final quantitative analysis (16–30).

### Study characteristics

The characteristics of the studies included in the meta-analysis are presented in **Table 1**. The 15 trials (16–30) included 1,306 participants, of whom 685 underwent prehabilitation strategies and 621 received standard care or rehabilitation only. The average age of patients in both groups was 70 years. Eight, three, and four studies included patients undergoing multimodal prehabilitation (17, 19–21, 23, 26, 27, 30), exercise (16, 25, 29), and nutrition optimization, respectively (18, 22, 24, 28).

**Table 1:** Characteristics of included studies comparing prehabilitation versus standard care or rehabilitation among patients undergoing colorectal surgery.

Study	Year	Country	Study design	Prehabilitation strategies	Control type	Numbers of participants		Age	
						Intervention	Control	Intervention	Control
Berkel (16)	2021	The Netherlands	RCT	Exercise	Usual care	28	29	74	73
Bousquet-Diona (17)	2018	Canada	RCT	Multimodal	Rehabilitation	37	26	74	71
Burden (18)	2011	UK	RCT	Nutrition	Usual care	54	62	64.5	65.3
Carli (19)	2020	Canada	RCT	Multimodal	Rehabilitation	55	55	78	82
Chia (20)	2015	Singapore	Prospective study	Multimodal	Usual care	57	60	79	80.5
Fulop (21)	2020	Hungary	RCT	Multimodal	Usual care	77	72	70	70
Gilbert (22)	2021	France	Stepped wedge trial	Nutrition	Usual care	74	73	80.5	79.2
Gillis (23)	2014	Canada	RCT	Multimodal	Rehabilitation	38	39	65.7	66
Gillis (24)	2015	Canada	RCT	Nutrition	Usual care	22	21	67.6	69.1
Hernon (25)	2021	UK	RCT	Exercise	Usual care	137	63	67.1	69.1
Li (26)	2012	Canada	RCT	Multimodal	Usual care	42	45	67.4	66.4
López-Rodríguez-Arias (27)	2021	Spain	RCT	Multimodal	Usual care	10	10	66.5	66
MacFie (28)	2000	UK	RCT	Nutrition	Usual care	24	25	68	64
Northgraves (29)	2019	UK	RCT	Exercise	Usual care	10	11	64.1	63.5
van Rooijen (30)	2019	The Netherlands	RCT	Multimodal	Usual care	20	30	75	71

### Risk of bias in the included studies

The risk of bias is summarized in the Supplementary Material (**Figure S1**). One of the 15 included studies was a prospective study (20), which was not included in the subsequent assessment. Of the remaining 14 trials, 1 was open-labeled (22), 12 (16–19, 21–25, 27–29) used appropriate random sequence generation, and 10 (16–19, 21–25, 27, 29) used allocation concealment. Only two trials used double-blinded methods (16, 24), five trials were single-blinded (17–19, 23, 25), and one trial was unblinded (28). Others were open-label or failed to state blinding methods. Seven of the RCTs reported using blinded assessors for outcome indicators. No reporting bias was observed in this study. As the studies in abstract form and meeting reports were not eligible in this meta-analysis, no other bias was considered. Overall, 10 studies (16–19, 21, 23–25, 27, 29) were deemed high quality, whereas 4 (22, 26, 28, 30) were graded as having a high risk of bias.

### Effect of prehabilitation on overall complications

**Figure 2:**
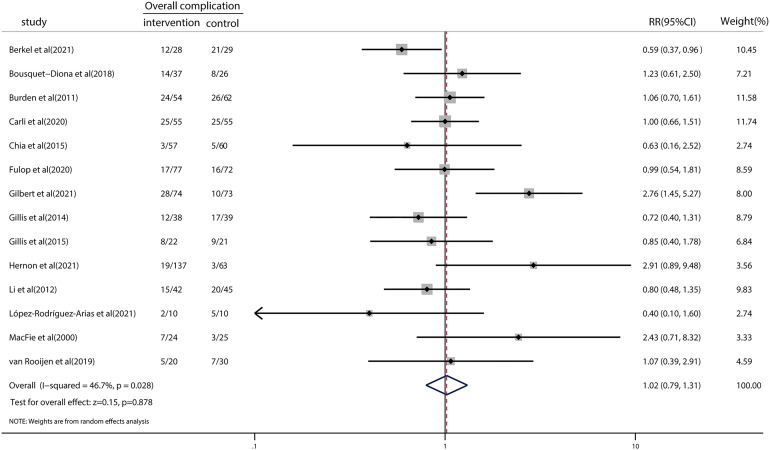
Forest plot for overall complications after colorectal surgery with or without preoperative prehabilitation strategies.

We examined the effects of prehabilitation on postoperative complications. The risk ratio in overall complications was 1.02 (95% CI = 0.79–1.31; *p* = 0.878; **Figure 2**), indicating no significant reduction in the risk of clinically important postoperative complications following prehabilitation. There was a moderate level of heterogeneity (*I* ^2^ = 46.7%; *p* = 0.028). We then performed a meta-regression to explore the potential sources of heterogeneity (**Table S2**). The results indicated that year of publication (*p* = 0.718), age (*p* = 0.829), type of control (*p* = 0.877), and geographical location (*p* = 0.255) did not significantly influence the results of meta-analysis regarding the overall complications. Furthermore, the detailed type of prehabilitation strategies was assessed by subgroup analysis for exercise, nutrition, or trimodal prehabilitation (**Figure S2**). Subgroup analysis results demonstrated that the risk ratios for postoperative complications in studies concerning exercise, nutrition, and trimodal prehabilitation were 1.22 (95% CI = 0.22–6.86), 1.47 (95% CI = 0.81–2.66), and 1.02 (95% CI = 0.79–1.31), respectively. No significant differences were found between subgroups.

**Figure 3:**
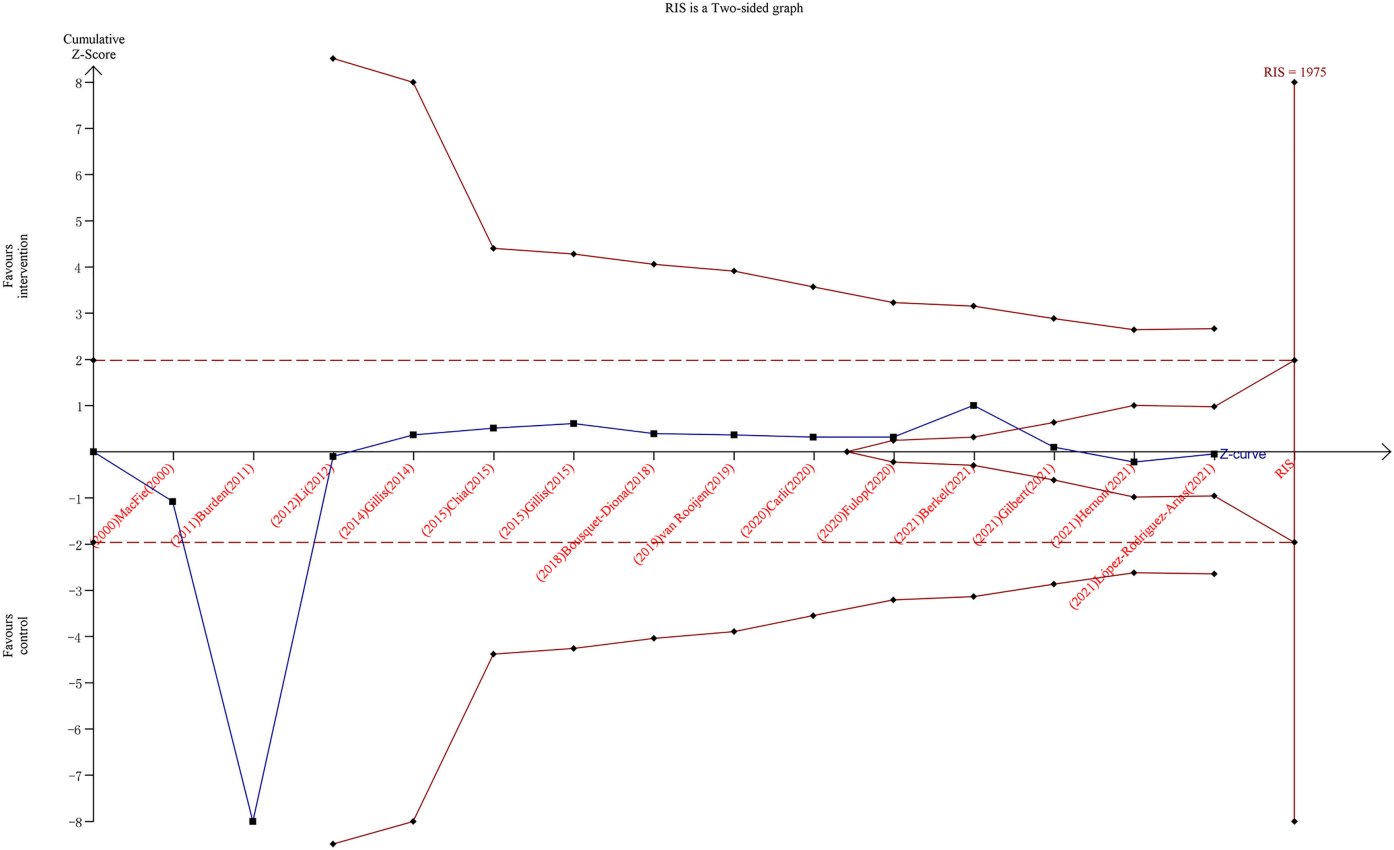
Trial sequential analysis for overall complications after colorectal surgery with or without preoperative prehabilitation strategies. The blue z-curve was drawn by applying a random-effects model.

As shown in the TSA (**Figure 3**), the RIS was calculated as 1,975 patients for overall complications, whereas the *z*-curve crossed the adjusted TSA boundary favoring the intervention and control groups, indicating no need for further trials to validate the conclusions.

### Effect of prehabilitation on LOS

**Figure 4:**
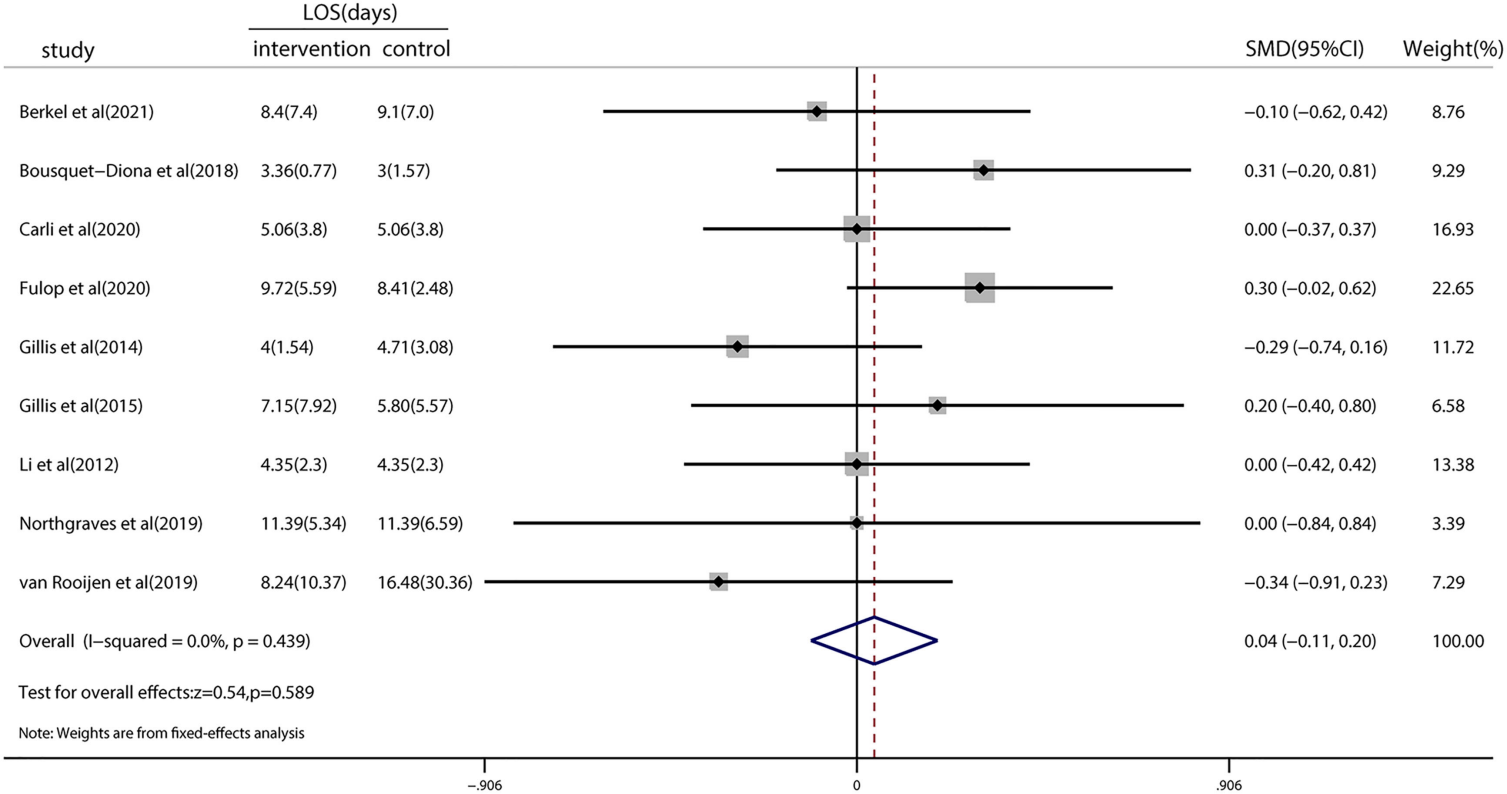
Forest plot for the length of hospital stay after colorectal surgery with or without preoperative prehabilitation strategies.

Nine studies investigated the LOS, and the pooled results showed no significant reduction (SMD = 0.04; 95% CI = −0.11 to 0.20; *p* = 0.589; **Figure 4**). Heterogeneity (*I* ^2^ < 0.001%; *p* = 0.439) among the studies reporting this outcome was low. TSA revealed that the *z*-curve did not cross traditional boundaries. However, the boundary RIS was not available because of insufficient information use (3.65%). A detailed graph is shown in **Figure S3**.

### Effect of prehabilitation on functional capacity

Four studies examined the effect of prehabilitation on functional capacity as measured by the 6MWT. There was no significant difference in functional capacity at 4 weeks (SMD = 0.16; 95% CI = −0.06 to 0.38; *p* = 0.144; **Figure S4**) or 8 weeks postoperatively (SMD = 0.18; 95% CI = −0.21 to 0.56; *p* = 0.367; **Figure S5**).

### Publication bias and sensitivity analysis

We performed Harbord’s test to assess the publication bias of dichotomous data for the primary outcome. Egger’s and Begg’s tests were conducted to evaluate the publication bias of continuous data for the primary outcome. The result of Harbord’s test was 0.291 for the overall complications. Regarding LOS, the results of Egger’s test (0.375) and Begg’s test (0.754) further revealed no publication bias. Visual inspection of the funnel plots did not raise concerns about publication bias (**Figures S6, S7**). The effect estimation of sensitivity analysis showed that the results were stable, regardless of pooled complications or pooled LOS (**Figures S8, S9**).

### Certainty of evidence

The certainty of evidence assessment of the primary outcomes is summarized in **Table 2**. The evidence was rated as moderate for overall complications, LOS, and 6MWT at 8 weeks postoperatively and high for 6MWT at 4 weeks postoperatively. The outcomes for overall complications and LOS were downgraded to one level for risk of bias. The 6MWT at 8 weeks postoperatively was also downgraded to one level owing to concerns regarding the risk of inconsistency.

**Table 2:** Summary of findings.

Outcomes	Participants (studies)	Risk of bias	Inconsistency	Indirectness	Imprecision	Publication bias	RR/SMD 95% CI	Overall quality of evidence (GRADE)
Overall complications	1,285 (13 RCTs and one prospective study)	Serious^1^	No serious inconsistency	No serious indirectness	No serious imprecision	No serious publication bias	RR 1.02 95% CI (0.79, 1.31)	⊕⊕⊕◯ MODERATE^1^
LOS	600 (9 RCTs)	Serious^1^	No serious inconsistency	No serious indirectness	No serious imprecision	No serious publication bias	SMD 0.04 95% CI (−0.11, 0.2)	⊕⊕⊕◯ MODERATE^1^
6MWT at 4 weeks after surgery	322 (3 RCTs)	No serious risk of bias	No serious inconsistency	No serious indirectness	No serious imprecision	No serious publication bias	SMD 0.16 95% CI (−0.06, 0.38)	⊕⊕⊕⊕ HIGH
6MWT at 8 weeks after surgery	289 (3 RCTs)	No serious risk of bias	Serious^2^	No serious indirectness	No serious imprecision	No serious publication bias	SMD 0.18 95% CI (−0.21, 0.56)	⊕⊕⊕◯ MODERATE^2^

^1^Risk of bias existed in two to three trials.

^2^I^2^ > 50%, indicating that the inconsistency existed.

## Discussion

This meta-analysis, which included 15 trials and 1,306 patients, compared prehabilitation intervention with standard care and rehabilitation in patients undergoing colorectal surgery. The main findings showed no significant differences in postoperative complications, LOS, and 6MWT. To analyze the postoperative complications, a meta-regression was performed based on the possible moderators (year of publication, age, type of control, and geographical location), with no statistic heterogeneity reported. The subgroup analysis of the intervention strategies used, including exercise, nutrition, and multimodal prehabilitation, was also conducted. Similarly, no significant differences were observed among the subgroups.

Poor prognosis after major surgery has been emphasized increasingly by clinicians. Major surgery is thus often compared to a marathon, i.e., only well-prepared patients can endure it (31, 32). Patients who undergo colorectal surgery are generally older and have multiple morbidities. They may also have a high risk of frailty, with a decreased physiological reserve and anti-stress ability, which could trigger adverse outcomes and lower postoperative quality of life (33–35). Therefore, preoperative improvement is crucial for these patients. However, the current preoperative workup mainly focuses on identifying the risk factors, and less attention is paid to improving preoperative reserves (6).

Prehabilitation is an emerging strategy that aims to optimize patients for surgical procedures (36, 37). However, limited solid evidence proved the effects of prehabilitation in patients undergoing colorectal surgery, and detailed optimization strategies remain challenging. Previous systematic reviews had controversial conclusions regarding the effect of prehabilitation on patients undergoing colorectal surgery. In 2016, Bruns and colleagues reported that prehabilitation can improve the physical condition of patients for colorectal surgery, although no significant reduction in complications or LOS was observed (8). Moran et al. reported that prehabilitation appears to be beneficial in decreasing the incidence of postoperative complications after the intra-abdominal operation, only four of nine enrolled studies included patients undergoing colorectal surgery. The authors mainly focused on exercise programs and the methodologic quality of included studies was relatively low (38). In 2018, a meta-analysis by Gillis et al. documented that nutritional prehabilitation with or without exercise significantly reduced LOS by 2 days in patients undergoing colorectal surgery (9). Their results on LOS are inconsistent with ours. However, we included six studies published after 2018, thus making our current analysis much more comprehensive. In 2020, Lambert and others performed a meta-analysis on prehabilitation of patients undergoing hepatobiliary, colorectal, and upper gastrointestinal cancer surgeries (10). Their results demonstrated that prehabilitation was associated with a shorter LOS but had no effect on functional capacity, postoperative complications, or mortality. A recent Cochrane review, including three RCTs and a total of 250 patients, indicated that prehabilitation may improve functional capacity postoperatively and result in fewer complications, while no difference was reported regarding LOS (39). Our findings are partly in line with those of Lambert and others; however, our study provides a more comprehensive analysis of colorectal surgery as it included 1,306 patients from 14 RCTs and one prospective study. The certainty of evidence generated from our meta-analysis was also rated as moderate for the primary and secondary outcomes. In fact, the reasons for the lack of significant differences in postoperative complications, LOS, and 6MWT results are complex and multifactorial. It should be noted that most of the included studies were conducted after 2011 when the ERAS protocol was implemented. Studies have shown that ERAS alone significantly improved the short-term surgical outcomes of patients undergoing colorectal surgery (40); thus, the effect of prehabilitation might be underestimated if assessed within an ERAS population. In our study, no prehabilitation strategy was found in the control group of the included trials. Thus, the reason why prehabilitation did not significantly affect the outcomes of patients undergoing colorectal surgery in our meta-analysis may not be attributed to any optimization in the control group.

In our study, we used TSA to further evaluate the endpoints of overall complications. Type I and II errors were set at 5% and 20%, respectively. The incidence of controls was 40% based on our enrolled data, and a 20% relative risk reduction was assigned to calculate the required information size. Following these settings, the optimal number of samples was 1,975, and 1,306 samples were included in this meta-analysis. The cumulative *z*-curve crossed the adjusted TSA boundary, favoring the intervention and control groups. This finding demonstrates that further trials to confirm this negative result are unnecessary. Thus, based on the current evidence, we can assume that prehabilitation has no advantages in terms of overall complications. We also searched the aforementioned database of clinical studies conducted worldwide, and we observed that many clinical trials on this theme are ongoing or complete. For example, over a dozen studies are registered at clinicaltrials.gov and at the stage of participant recruitment, to explore the effects of prehabilitation on patients undergoing colorectal surgery with various prehabilitation strategies. Unfortunately, no results are available currently, and whether these trials may change the conclusion of our current meta-analysis remains unknown.

This systematic review benefits from robust methods in keeping with the established guidelines (41), including a registered protocol. Three previous meta-analyses have shown that prehabilitation might be a promising intervention to improve certain adverse outcomes after surgery (e.g., lung resections, major abdominal surgery, and cardiac surgery) (42–44). Our study mainly focused on colorectal surgery and no significant benefits were observed. The results may only target patients undergoing colorectal surgery, and may not be applicable to other kinds of operations. Besides, our study had some limitations. First, we included one prospective study, which was bound to increase heterogeneity. Second, owing to insufficient information on mortality and confounders (e.g., age, tumor stage, radiotherapy, and chemotherapy) that may influence mortality, the effect of prehabilitation on postoperative mortality was not examined. Third, few studies on exercise and psychological prehabilitation have been conducted, making it difficult to fully analyze their effects. Fourth, the sample sizes of the included studies were small, reducing the confidence in the reported outcomes. Based on these limitations, more optimal and high-quality research is required in the near future. A recent umbrella review of 55 systematic reviews demonstrated that prehabilitation may yet improve postoperative outcomes with low certainty (45). The authors conducted the analysis with populations undergoing various surgical procedures, with cancer surgeries (22 of 55) being the most common focus of included reviews. However, including overlapping trials into the umbrella review can cause double counting of evidence, contributing a certain degree of limitation. Their work also highlights the optimization of trial execution to increase the certainty of the effectiveness of prehabilitation.

## Conclusions

In conclusion, this study demonstrated that prehabilitation of patients undergoing colorectal surgery does not significantly affect postoperative complications, LOS, and 6MWT. Thus, prehabilitation strategies may not be beneficial in colorectal surgery, and there is limited direct evidence supporting the recommendation of prehabilitation for patients undergoing colorectal surgery. Whether it is necessary to continue this program deserves further consideration. High-quality clinical trials for patients with a higher risk of postoperative complications are warranted, and targeted and intensive individualized prehabilitation plans are required to guide the best clinical practice.

## Data availability statement

The original contributions presented in the study are included in the article/**Supplementary Material**. Further inquiries can be directed to the corresponding author.

## Author contributions

LB, ZJ, and XZ designed the study. XZ, SW, WJ, HW, and KZ made contributions to the conduct of the study. XZ, SW, WJ, and ZJ made the data analysis. XZ and LB were the major contributors to writing the first draft of the manuscript. All authors listed contributed to reviewing and approving the final version of the manuscript.

## Funding

This work was supported by the Shanghai Science and Technology Committee Rising-Star Program (grant number 19QA1408500) and the “234 Discipline Construction Climbing Plan” of the Changhai Hospital, Naval Medical University (grant number 2020YXK053).

## Conflict of interest

The authors declare that the research was conducted in the absence of any commercial or financial relationships that could be construed as a potential conflict of interest.

## Publisher’s note

All claims expressed in this article are solely those of the authors and do not necessarily represent those of their affiliated organizations, or those of the publisher, the editors and the reviewers. Any product that may be evaluated in this article, or claim that may be made by its manufacturer, is not guaranteed or endorsed by the publisher.

## References

[1] Sung H Ferlay J Siegel RL Laversanne M Soerjomataram I Jemal A . Global cancer statistics 2020: GLOBOCAN estimates of incidence and mortality worldwide for 36 cancers in 185 countries. CA A Cancer J Clin (2021) 71:209–49. doi: 10.3322/caac.21660 33538338

[2] Ripollés-Melchor J Ramírez-Rodríguez JM Casans-Francés R Aldecoa C Abad-Motos A Logroño-Egea M . Association between use of enhanced recovery after surgery protocol and postoperative complications in colorectal surgery: the postoperative outcomes within enhanced recovery after surgery protocol (POWER) study. JAMA Surg (2019) 154:725–36. doi: 10.1001/jamasurg.2019.0995 PMC6506896 31066889

[3] Gillis C Ljungqvist O Carli F . Prehabilitation, enhanced recovery after surgery, or both? a narrative review. Br J Anaesth (2022) 128:434–48. doi: 10.1016/j.bja.2021.12.007 35012741

[4] Morris PE Berry MJ Files DC Thompson JC Hauser J Flores L . Standardized rehabilitation and hospital length of stay among patients with acute respiratory failure: A randomized clinical trial. JAMA (2016) 315:2694–702. doi: 10.1001/jama.2016.7201 PMC6657499 27367766

[5] Wynter-Blyth V Moorthy K . Prehabilitation: preparing patients for surgery. BMJ (2017) 358:j3702. doi: 10.1136/bmj.j3702 28790033

[6] Howard R Yin YS McCandless L Wang S Englesbe M Machado-Aranda D . Taking control of your surgery: impact of a prehabilitation program on major abdominal surgery. J Am Coll Surg (2019) 228:72–80. doi: 10.1016/j.jamcollsurg.2018.09.018 30359831 PMC6309718

[7] Trépanier M Minnella EM Paradis T Awasthi R Kaneva P Schwartzman K . Improved disease-free survival after prehabilitation for colorectal cancer surgery. Ann Surg (2019) 270:493–501. doi: 10.1097/SLA.0000000000003465 31318793

[8] Bruns ER van den Heuvel B Buskens CJ van Duijvendijk P Festen S Wassenaar EB . The effects of physical prehabilitation in elderly patients undergoing colorectal surgery: a systematic review. Colorectal Dis (2016) 18:O267–77. doi: 10.1111/codi.13429 27332897

[9] Gillis C Buhler K Bresee L Carli F Gramlich L Culos-Reed N . Effects of nutritional prehabilitation, with and without exercise, on outcomes of patients who undergo colorectal surgery: A systematic review and meta-analysis. Gastroenterol New York (2018) 155:391–410.e4. doi: 10.1053/j.gastro.2018.05.012 29750973

[10] Lambert JE Hayes LD Keegan TJ Subar DA Gaffney CJ . The impact of prehabilitation on patient outcomes in hepatobiliary, colorectal, and upper gastrointestinal cancer surgery: a PRISMA-accordant meta-analysis. Ann Surg (2021) 274:70–7. doi: 10.1097/SLA.0000000000004527 33201129

[11] Moher D Liberati A Tetzlaff J Altman DG PRISMA Group . Preferred reporting items for systematic reviews and meta-analyses: the PRISMA statement. Ann Intern Med (2009) 151:264–9, W64. doi: 10.7326/0003-4819-151-4-200908180-00135 19622511

[12] Jin ZC Zhou XH He J . Statistical methods for dealing with publication bias in meta-analysis. Stat Med (2015) 34:343–60. doi: 10.1002/sim.6342 25363575

[13] Guyatt GH Oxman AD Kunz R Vist GE Falck-Ytter Y Schünemann HJ . What is “quality of evidence” and why is it important to clinicians? BMJ (2008) 336:995–8. doi: 10.1136/bmj.39490.551019.BE PMC2364804 18456631

[14] Agarwala P Salzman SH . Six-minute walk test: clinical role, technique, coding, and reimbursement. Chest (2020) 157:603–11. doi: 10.1016/j.chest.2019.10.014 PMC7609960 31689414

[15] Wan X Wang W Liu J Tong T . Estimating the sample mean and standard deviation from the sample size, median, range and/or interquartile range. BMC Med Res Methodol (2014) 14:135. doi: 10.1186/1471-2288-14-135 25524443 PMC4383202

[16] Berkel AEM Bongers BC Kotte H Weltevreden P de Jongh FHC Eijsvogel MMM . Effects of community-based exercise prehabilitation for patients scheduled for colorectal surgery with high risk for postoperative complications: Results of a randomized clinical trial. Ann Surg (2022) 275:e299–306. doi: 10.1097/SLA.0000000000004702 PMC8746915 33443905

[17] Bousquet-Dion G Awasthi R Loiselle SÈ Minnella EM Agnihotram RV Bergdahl A . Evaluation of supervised multimodal prehabilitation programme in cancer patients undergoing colorectal resection: a randomized control trial. Acta Oncol (2018) 57:849–59. doi: 10.1080/0284186X.2017.1423180 29327644

[18] Burden ST Hill J Shaffer JL Campbell M Todd C . An unblinded randomised controlled trial of preoperative oral supplements in colorectal cancer patients. J Hum Nutr Diet (2011) 24:441–8. doi: 10.1111/j.1365-277X.2011.01188.x 21699587

[19] Carli F Bousquet-Dion G Awasthi R Elsherbini N Liberman S Boutros M . Effect of multimodal prehabilitation vs postoperative rehabilitation on 30-day postoperative complications for frail patients undergoing resection of colorectal cancer: A randomized clinical trial. JAMA Surg (2020) 155:233–42. doi: 10.1001/jamasurg.2019.5474 PMC6990653 31968063

[20] Chia CLK Mantoo SK Tan KY . ‘Start to finish trans-institutional transdisciplinary care’: a novel approach improves colorectal surgical results in frail elderly patients. Colorectal Dis (2016) 18:O43–50. doi: 10.1111/codi.13166 26500155

[21] Fulop A Lakatos L Susztak N Szijarto A Banky B . The effect of trimodal prehabilitation on the physical and psychological health of patients undergoing colorectal surgery: a randomised clinical trial. Anaesthesia (2021) 76:82–90. doi: 10.1111/anae.15215 32761611

[22] Gilbert T Bernard L Alexandre M Bin-Dorel S Villeneuve L Decullier E . Impact of a geriatric intervention to improve screening and management of undernutrition in older patients undergoing surgery for colorectal cancer: results of the ANC stepped-wedge trial. Nutrients (2021) 13:2347. doi: 10.3390/nu13072347 34371859 PMC8308889

[23] Gillis C Li C Lee L Awasthi R Augustin B Gamsa A . Prehabilitation versus rehabilitation: a randomized control trial in patients undergoing colorectal resection for cancer. Anesthesiology (2014) 121:937–47. doi: 10.1097/ALN.0000000000000393 25076007

[24] Gillis CMR Loiselle SE Fiore JFPP Awasthi R Wykes L Liberman AS . Prehabilitation with whey protein supplementation on perioperative functional exercise capacity in patients undergoing colorectal resection for cancer: A pilot double-blinded randomized placebo-controlled trial. J Acad Nutr Diet (2016) 116:802–12. doi: 10.1016/j.jand.2015.06.007 26208743

[25] PREPARE-ABC Trial Collaborative . SupPoRtive exercise programmes for accelerating REcovery after major ABdominal cancer surgery trial (PREPARE-ABC): pilot phase of a multicentre randomised controlled trial. Colorectal Dis (2021) 23:3008–22. doi: 10.1111/codi.15856 34355484

[26] Li C Carli F Lee L Charlebois P Stein B Liberman AS . Impact of a trimodal prehabilitation program on functional recovery after colorectal cancer surgery: a pilot study. Surg Endosc (2013) 27:1072–82. doi: 10.1007/s00464-012-2560-5 23052535

[27] López-Rodríguez-Arias F Sánchez-Guillén L Aranaz-Ostáriz V Triguero-Cánovas D Lario-Pérez S Barber-Valles X . Effect of home-based prehabilitation in an enhanced recovery after surgery program for patients undergoing colorectal cancer surgery during the COVID-19 pandemic. Support Care Cancer (2021) 29:7785–91. doi: 10.1007/s00520-021-06343-1 PMC8225311 34169328

[28] MacFie J Woodcock NP Palmer MD Walker A Townsend S Mitchell CJ . Oral dietary supplements in pre- and postoperative surgical patients: a prospective and randomized clinical trial. Nutrition (2000) 16:723–8. doi: 10.1016/s0899-9007(00)00377-4 10978851

[29] Northgraves MJ Arunachalam L Madden LA Marshall P Hartley JE MacFie J . Feasibility of a novel exercise prehabilitation programme in patients scheduled for elective colorectal surgery: a feasibility randomised controlled trial. Support Care Cancer (2020) 28:3197–206. doi: 10.1007/s00520-019-05098-0 PMC7256075 31712950

[30] van Rooijen SJ Molenaar CJL Schep G van Lieshout RHMA Beijer S Dubbers R . Making patients fit for surgery: Introducing a four pillar multimodal prehabilitation program in colorectal cancer. Am J Phys Med Rehabil (2019) 98:888–96. doi: 10.1097/PHM.0000000000001221 31090551

[31] Martínez-Escribano C Arteaga Moreno F Pérez-López M Cunha-Pérez C Belenguer-Varea Á Cuesta Peredo D . Malnutrition and increased risk of adverse outcomes in elderly patients undergoing elective colorectal cancer surgery: A case-control study nested in a cohort. Nutrients (2022) 14:207. doi: 10.3390/nu14010207 35011082 PMC8746820

[32] STARSurg Collaborative . Impact of postoperative acute kidney injury in patients undergoing major gastrointestinal surgery on 1-year survival and renal outcomes: a national multicentre cohort study. BJS Open (2021) 5(6):zrab134. doi: 10.1093/bjsopen/zrab134 35029656 PMC8759520

[33] Boakye D Rillmann B Walter V Jansen L Hoffmeister M Brenner H . Impact of comorbidity and frailty on prognosis in colorectal cancer patients: A systematic review and meta-analysis. Cancer Treat Rev (2018) 64:30–9. doi: 10.1016/j.ctrv.2018.02.003 29459248

[34] Knight J Ayyash K Colling K Dhesi J Ewan V Danjoux G . A cohort study investigating the relationship between patient reported outcome measures and pre-operative frailty in patients with operable, non-palliative colorectal cancer. BMC Geriatr (2020) 20:311. doi: 10.1186/s12877-020-01715-4 32854632 PMC7453711

[35] Tamura K Matsuda K Fujita Y Iwahashi M Mori K Yamade N . Optimal assessment of frailty predicts postoperative complications in older patients with colorectal cancer surgery. World J Surg (2021) 45:1202–9. doi: 10.1007/s00268-020-05886-4 33392705

[36] Carli F Ferreira V . Prehabilitation: a new area of integration between geriatricians, anesthesiologists, and exercise therapists. Aging Clin Exp Res (2018) 30:241–4. doi: 10.1007/s40520-017-0875-8 29302796

[37] Durrand J Singh SJ Danjoux G . Prehabilitation. Clin Med (Lond) (2019) 19:458–64. doi: 10.7861/clinmed.2019-0257 PMC6899232 31732585

[38] Moran J Guinan E McCormick P Larkin J Mockler D Hussey J . The ability of prehabilitation to influence postoperative outcome after intra-abdominal operation: A systematic review and meta-analysis. Surgery (2016) 160(5):1189–201. doi: 10.1016/j.surg.2016.05.014 27397681

[39] Molenaar CJ van Rooijen SJ Fokkenrood HJ Roumen RM Janssen L Slooter GD . Prehabilitation versus no prehabilitation to improve functional capacity, reduce postoperative complications and improve quality of life in colorectal cancer surgery. Cochrane Database Syst Rev (2022) 5(5):CD013259. doi: 10.1002/14651858.CD013259.pub2 35588252 PMC9118366

[40] Ni X Jia D Chen Y Wang L Suo J . Is the enhanced recovery after surgery (ERAS) program effective and safe in laparoscopic colorectal cancer surgery? a meta-analysis of randomized controlled trials. J Gastrointest Surg (2019) 23:1502–12. doi: 10.1007/s11605-019-04170-8 30859422

[41] Higgins J Thomas J Chandler J Cumpston M Li T Page M . Cochrane handbook for systematic reviews of interventions version 6.2 (2021). Available at: https://training.cochrane.org/handbook/current.

[42] Cavalheri V Granger C . Preoperative exercise training for patients with non-small cell lung cancer. Cochrane Database Syst Rev (2017) 6:CD012020. doi: 10.1002/14651858.CD012020.pub2 28589547 PMC6481477

[43] Hughes MJ Hackney RJ Lamb PJ Wigmore SJ Christopher Deans DA Skipworth RJE . Prehabilitation before major abdominal surgery: A systematic review and meta-analysis. World J Surg (2019) 43:1661–8. doi: 10.1007/s00268-019-04950-y 30788536

[44] Zheng YT Zhang JX . Preoperative exercise and recovery after cardiac surgery: a meta-analysis. BMC Cardiovasc Disord (2020) 20:2. doi: 10.1186/s12872-019-01308-z 31914929 PMC6947961

[45] McIsaac DI Gill M Boland L Hutton B Branje K Shaw J . Prehabilitation in adult patients undergoing surgery: an umbrella review of systematic reviews. Br J Anaesth (2022) 128:244–57. doi: 10.1016/j.bja.2021.11.014 34922735

