# Supplementary material for: The effect of prehabilitation on the postoperative outcomes of patients undergoing colorectal surgery: A systematic review and meta-analysis
Source: Front Oncol. 2022 Jul 29;12:958261. doi: 10.3389/fonc.2022.958261 (PMC9372464; doi:10.3389/fonc.2022.958261)
Supplement: Supplementary file 1 [file DataSheet_1.docx]

**List of Supplemental Digital Content**

**Supplemental Table S1.** Detailed search strategy.

**Supplemental Fig S1.** Risk of bias summary and risk of bias graph

**Supplemental Table S2.** Meta-regression analysis of overall complications

**Supplemental Fig S2.** Forest plot for subgroup analysis of overall complications after colorectal surgery stratified with prehabilitation strategies.

**Supplemental Fig S3.** Trial sequential analysis for LOS after colorectal surgery with or without preoperative prehabilitation strategies.

**Supplemental Fig S4.** Forest plot for 6MWT at 4 weeks after colorectal surgery with or without preoperative prehabilitation strategies.

**Supplemental Fig S5.** Forest plot for 6MWT at 8 weeks after colorectal surgery with or without preoperative prehabilitation strategies.

**Supplemental Fig S6.** Funnel plot regarding overall complications.

**Supplemental Fig S7.** Funnel plot regarding LOS.

**Supplemental Fig S8.** Sensitivity analyses for overall complications

**Supplemental Fig S9.** Sensitivity analyses for LOS

**Supplemental Table S1.** Detailed search strategy.

1. perioperative care[mesh] OR perioperative[tw] OR peri-operative[tw] OR preoperative care[mesh] OR pre-operative[tw] OR preoperative[tw] OR prehab*[tw] OR pre-hab*[tw]
2. exercise[mesh] OR exercise*[tw] OR training[tw] OR physical*[tw] OR physiological*[tw] OR physiotherapy[tw]
3. nutrition therapy[mesh] OR diet[mesh] OR nutri*[tw] OR diet*[tw] OR supplement*[tw]
4. smoking cess*[tw] OR nicotine replacement[tw] OR alcohol cess*[tw] OR alcohol reduction[tw]
5. psychological*[tw] OR psychotherapy[tw] OR cognitive therapy[tw]
6. colorectal neoplasms[mesh] OR colorectal neoplasm*[tw] OR colorectal cancer*[tw] OR colorectal tumor*[tw] OR colorectal carcinoma*[tw]
7. colonic neoplasms[mesh] OR colonic neoplasm*[tw] OR colonic cancer*[tw] OR colon neoplasm*[tw] OR colon cancer*[tw]
8. rectal neoplasms[mesh] OR rectal neoplasm*[tw] OR rectal cancer*[tw] OR rectum neoplasm*[tw]OR rectum cancer*[tw]
9. (randomized controlled trial [pt] OR randomized controlled trial [pt] OR controlled clinical trial [pt] OR random*[tiab] OR placebo [tiab] OR trial [ti] OR clinical trials as topic [mesh] OR randomized controlled trial [mesh] ) NOT (animals [mh] NOT humans [mh] )
10. 2# OR 3# OR 4# OR 5#
11. 6# OR 7# OR 8#
12. 1# AND 10# AND 11# AND 9#


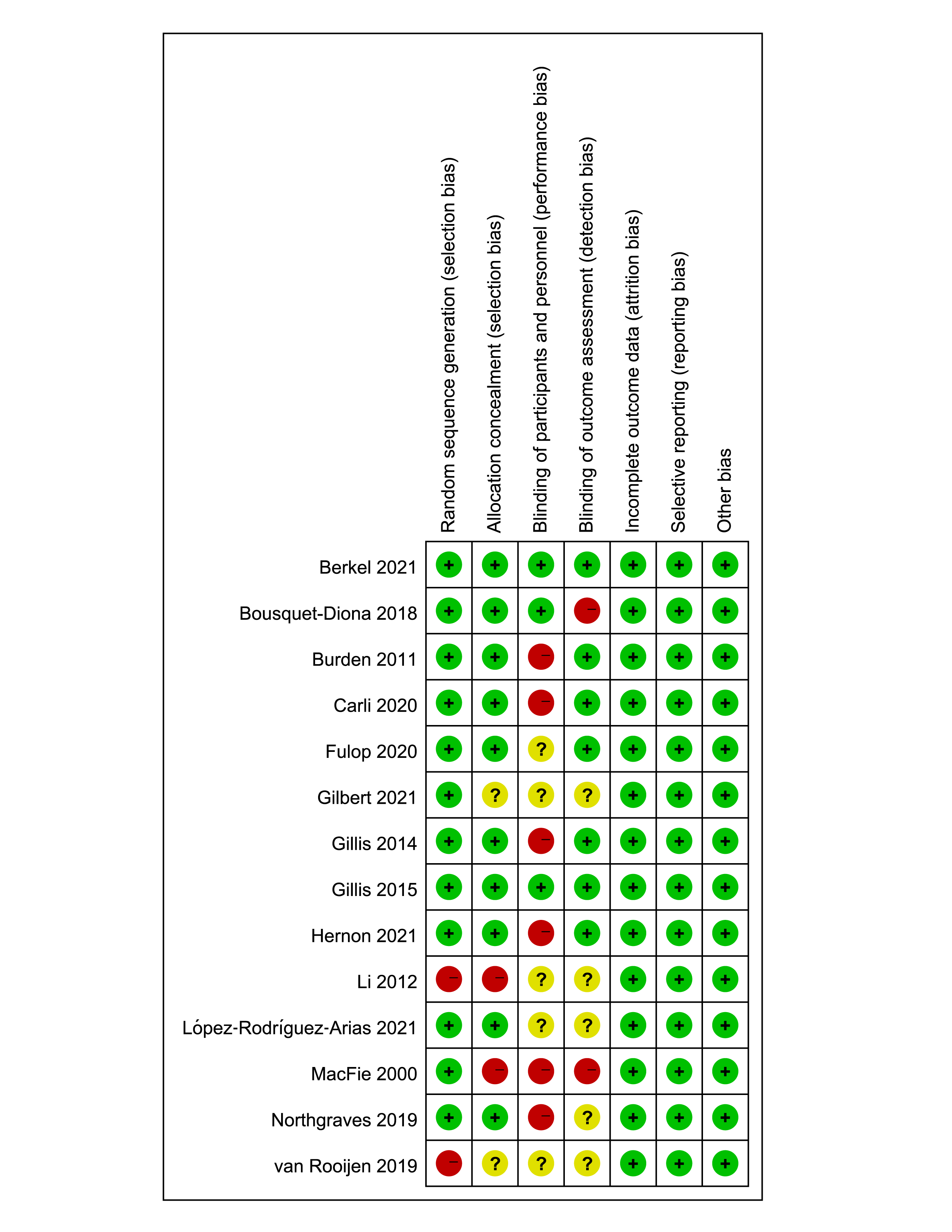


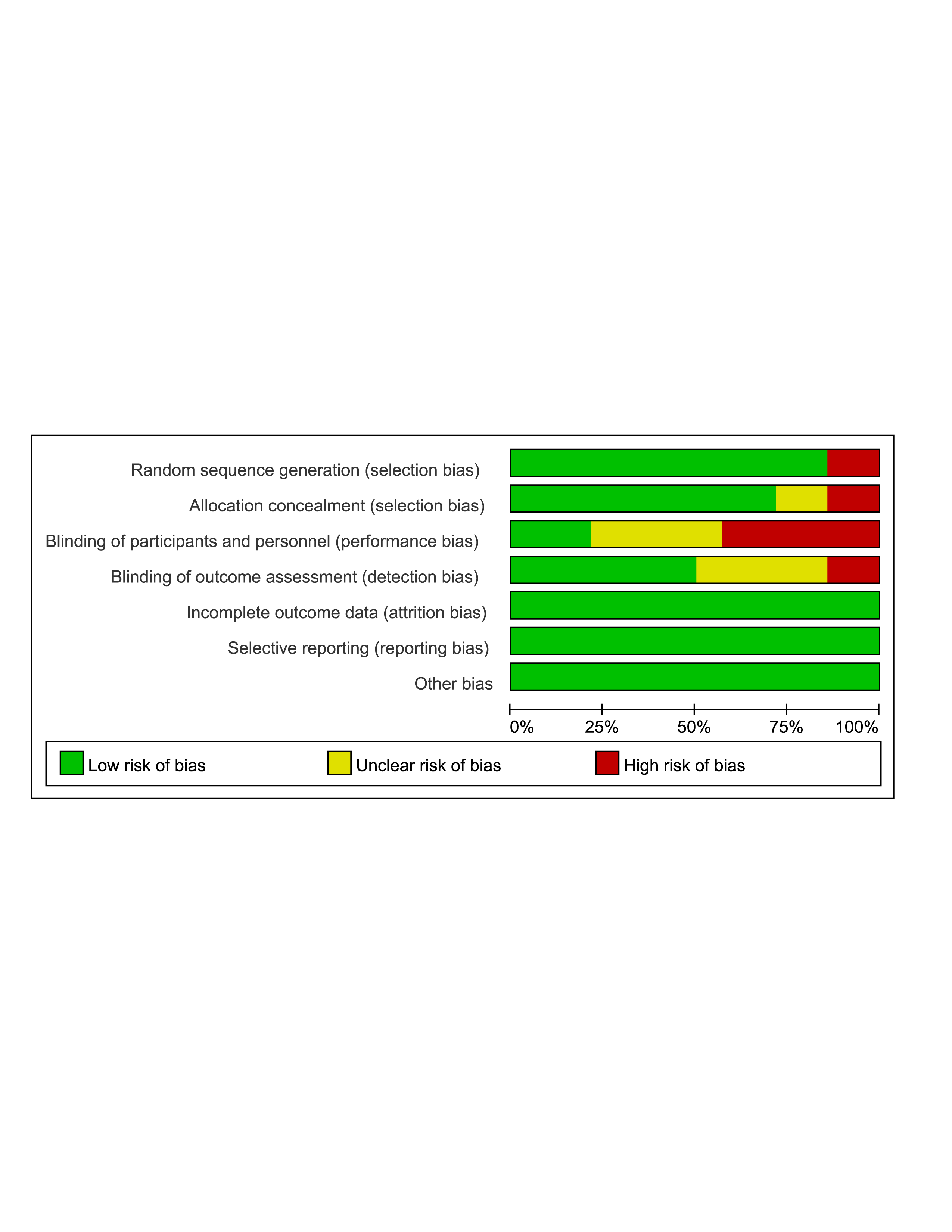


**Supplemental Fig S1.** Risk of bias summary and risk of bias graph

**Table S2.** Meta-regression analysis of overall complications

| **Variables** | **Number**  **of studies** | **Meta-regression**  **(P value)** | **Subgroup analysis** | |
| --- | --- | --- | --- | --- |
|  |  |  | **RR (95%CI)** | **I^2^** |
| **Year of publication** |  |  |  |  |
| 2000 | 1 | 0.718 | 2.43 (0.71, 8.32) | - |
| 2010~2015 | 5 |  | 0.88 (0.67, 1.14) | 0.0% |
| 2018~2021 | 8 |  | 1.12 (0.74, 1.7) | 65.0% |
| **Mean age** |  |  |  |  |
| ＜70 | 7 | 0.829 | 0.97 (0.69, 1.35) | 33.2% |
| ≥70 | 7 |  | 1.07 (0.72, 1.58) | 60.7% |
| **Type of control** |  |  |  |  |
| Usual care | 11 | 0.877 | 1.06 (0.75, 1.49) | 56.8% |
| Rehabilitation | 3 |  | 0.95 (0.70, 1.29) | 0.0% |
| **Geographical location** |  |  |  |  |
| America | 5 | 0.255 | 0.91 (0.71, 1.16) | 0.0% |
| Europe | 8 |  | 1.20 (0.76, 1.90) | 67.6% |
| Asia | 1 |  | 0.63 (0.16, 2.52) | - |


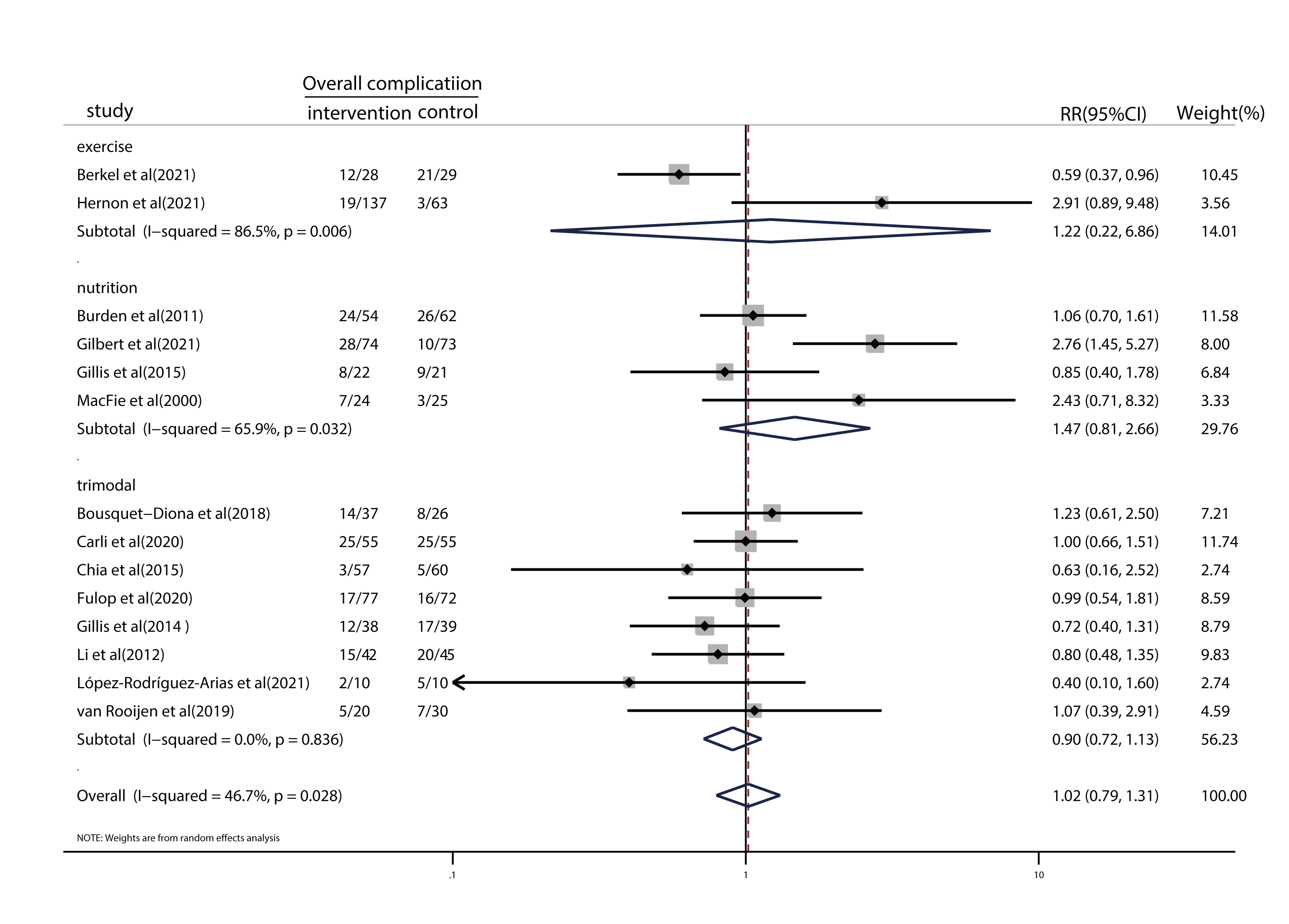


**Supplemental Fig S2.** Forest plot for subgroup analysis of overall complications after colorectal surgery stratified with prehabilitation strategies.


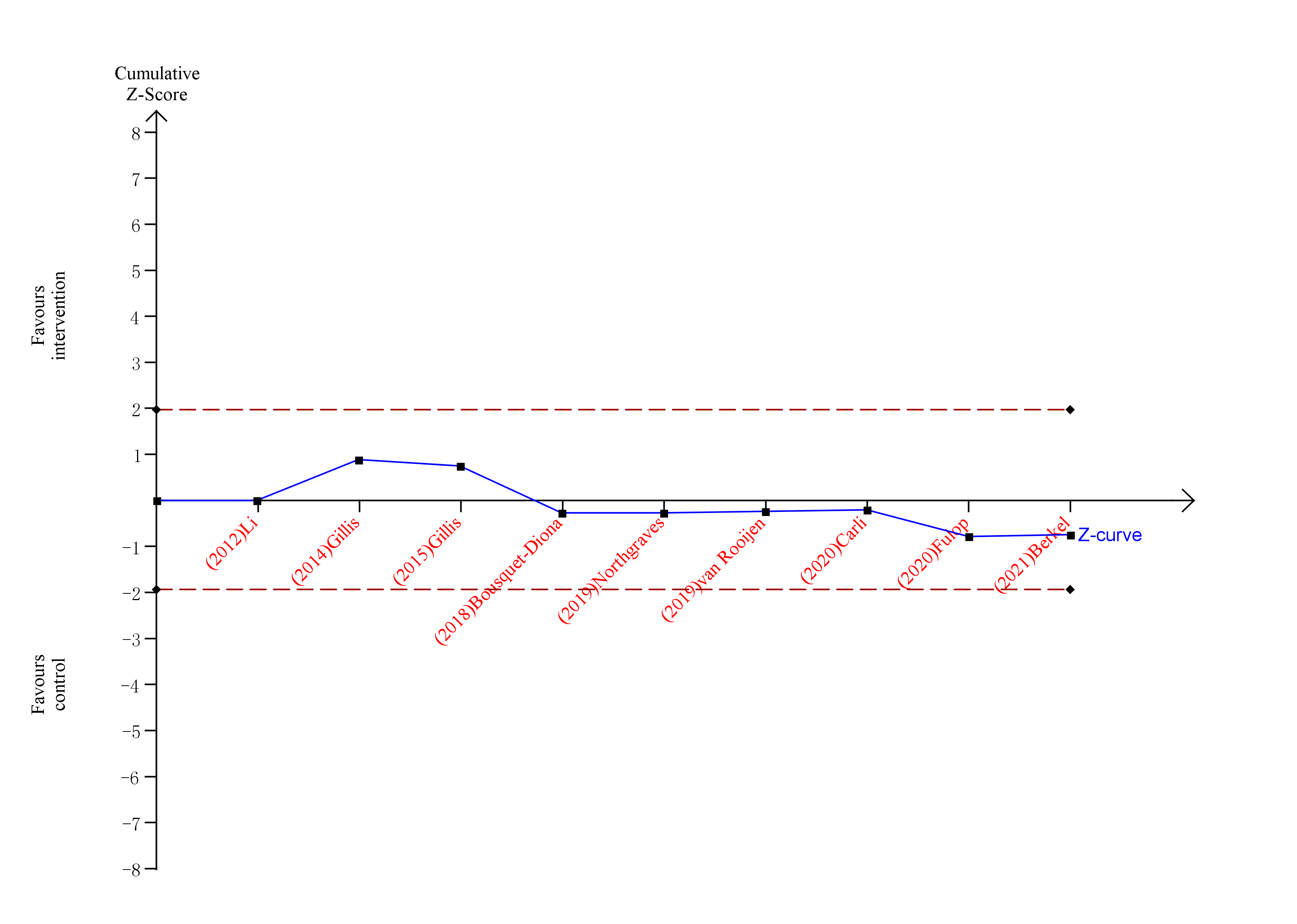


**Supplemental Fig S3.** Trial sequential analysis for LOS after colorectal surgery with or without preoperative prehabilitation strategies.


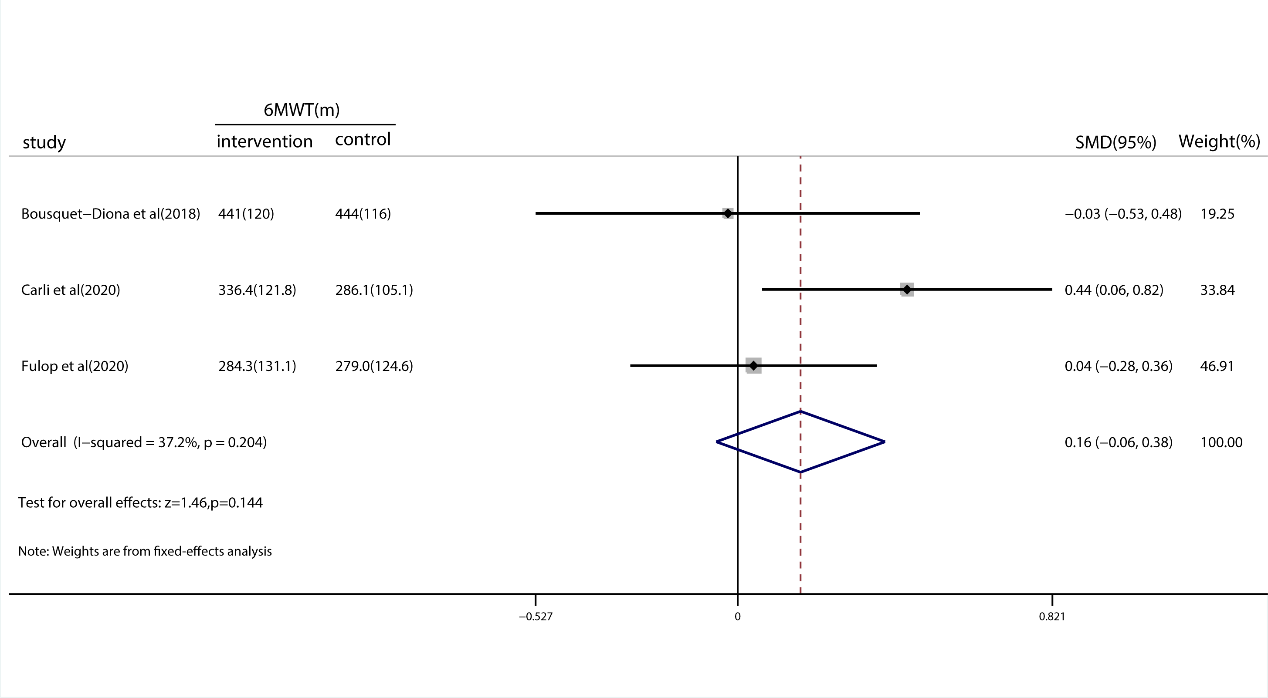


**Supplemental Fig S4.** Forest plot for 6MWT at 4 weeks after colorectal surgery with or without preoperative prehabilitation strategies.


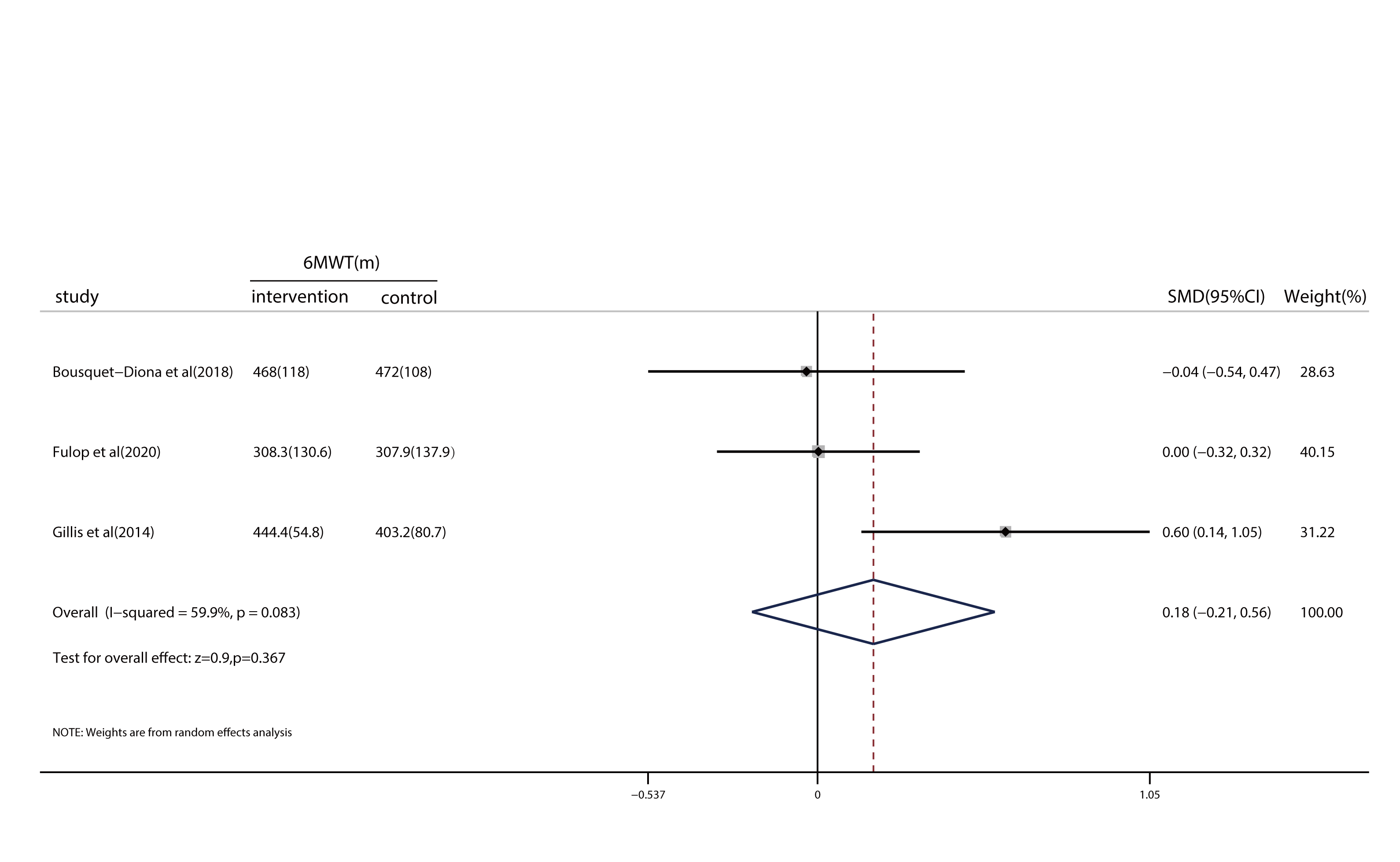


**Supplemental Fig S5.** Forest plot for 6MWT at 8 weeks after colorectal surgery with or without preoperative prehabilitation strategies.


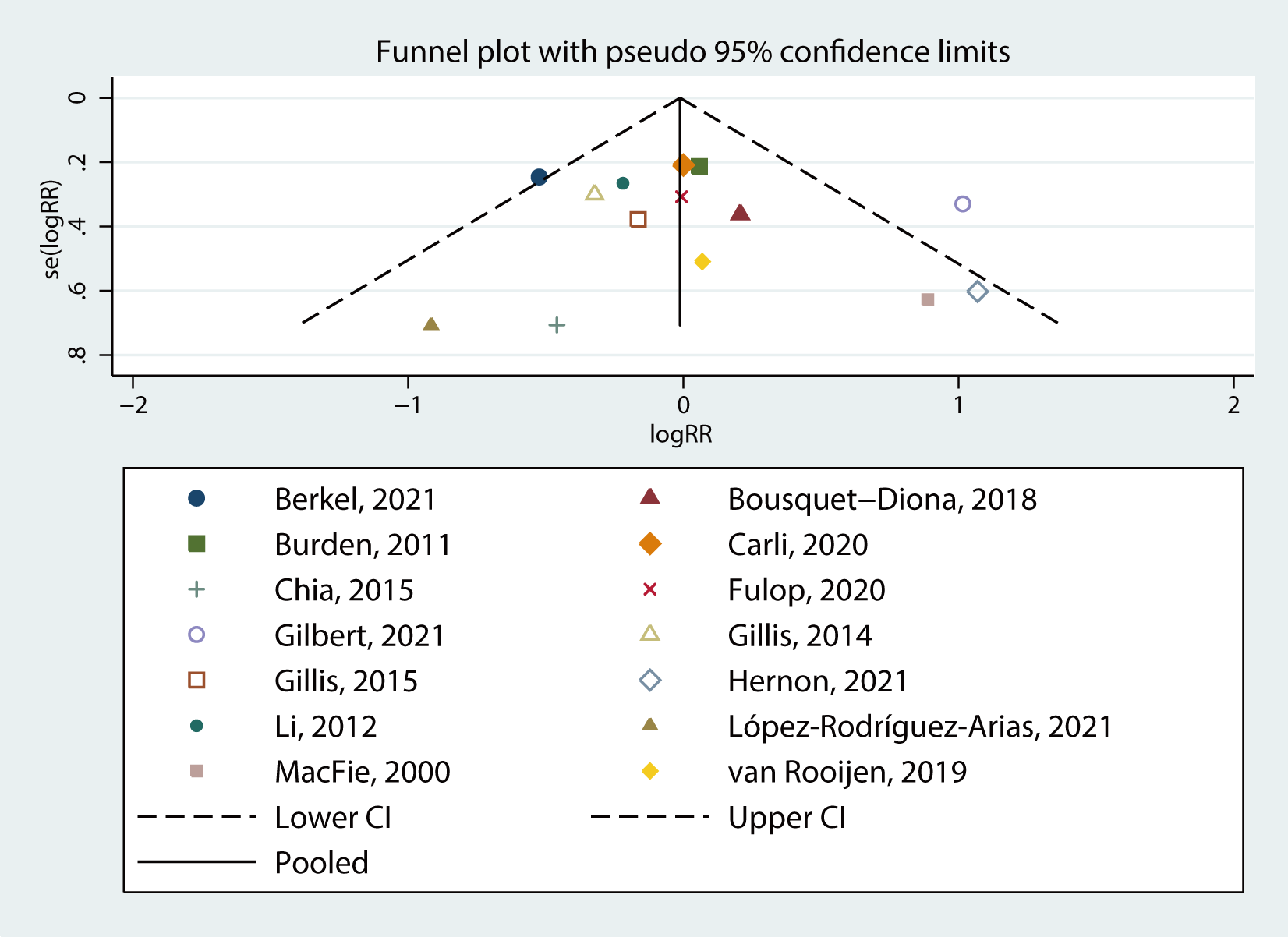


**Supplemental Fig S6.** Funnel plot regarding overall complications.


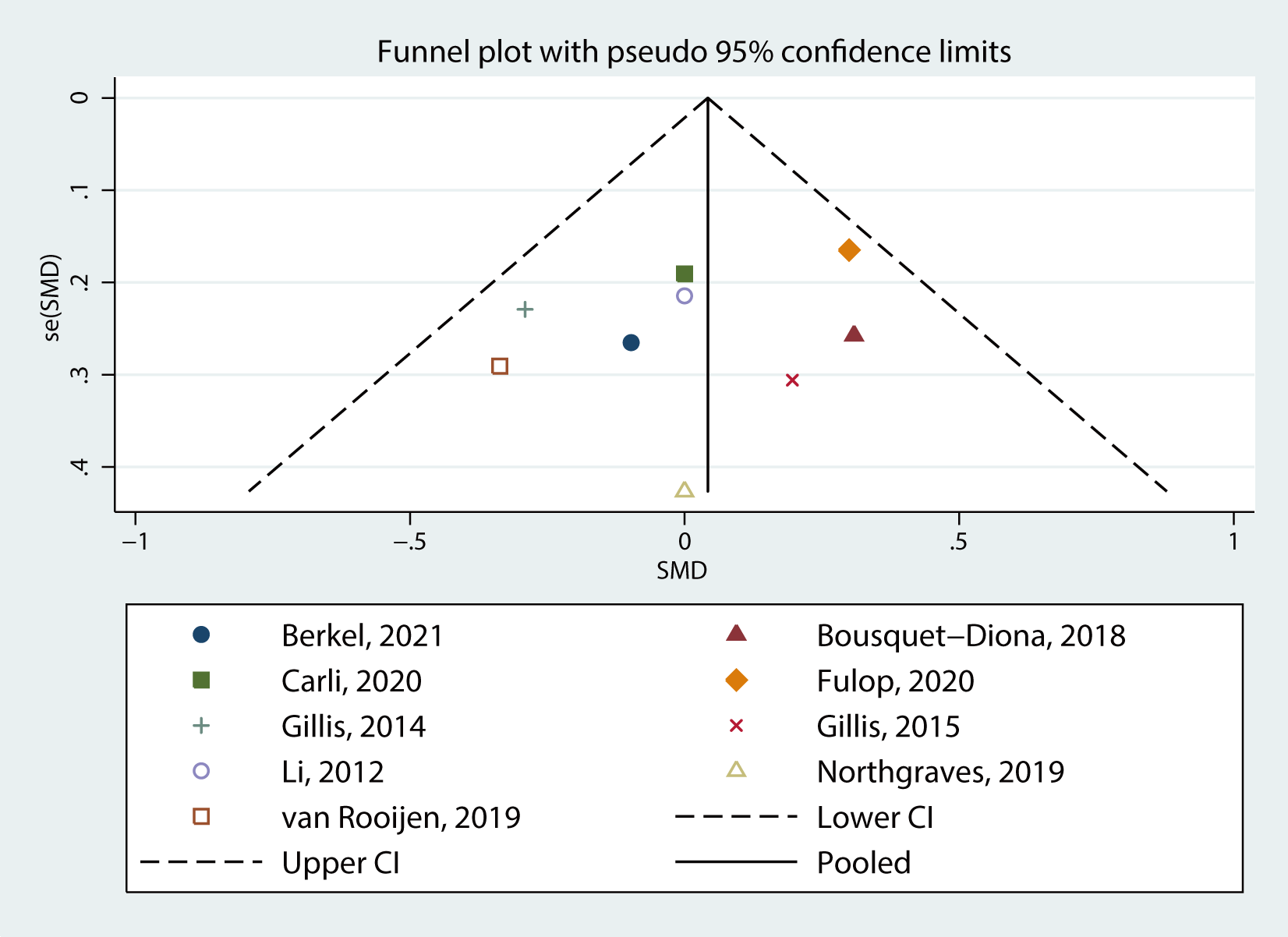


**Supplemental Fig S7.** Funnel plot regarding LOS.


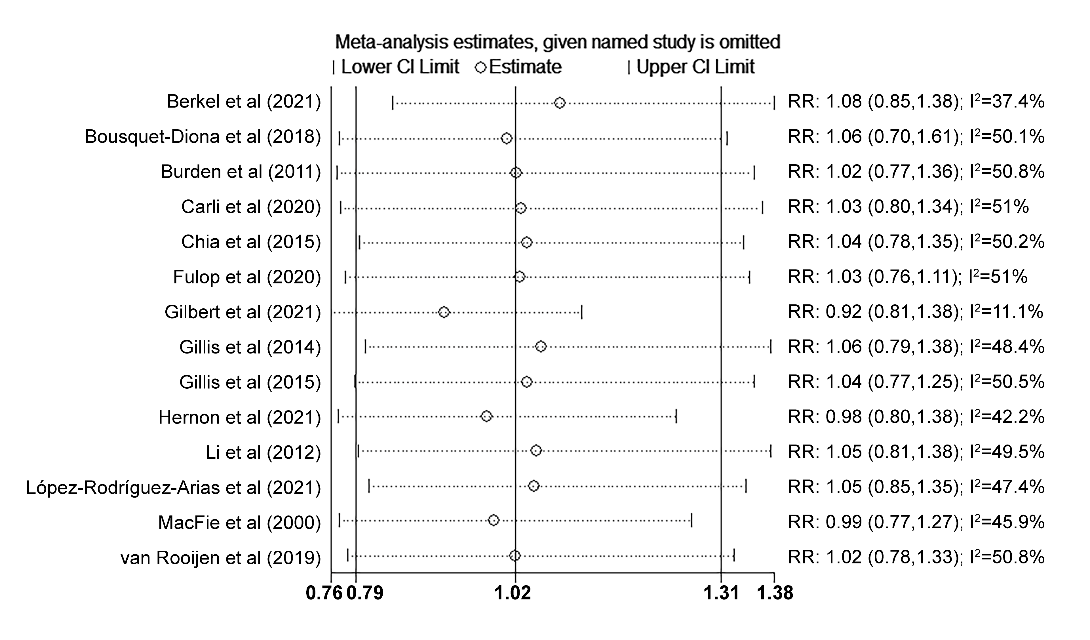


**Supplemental Fig S8.** Sensitivity analysis for overall complications


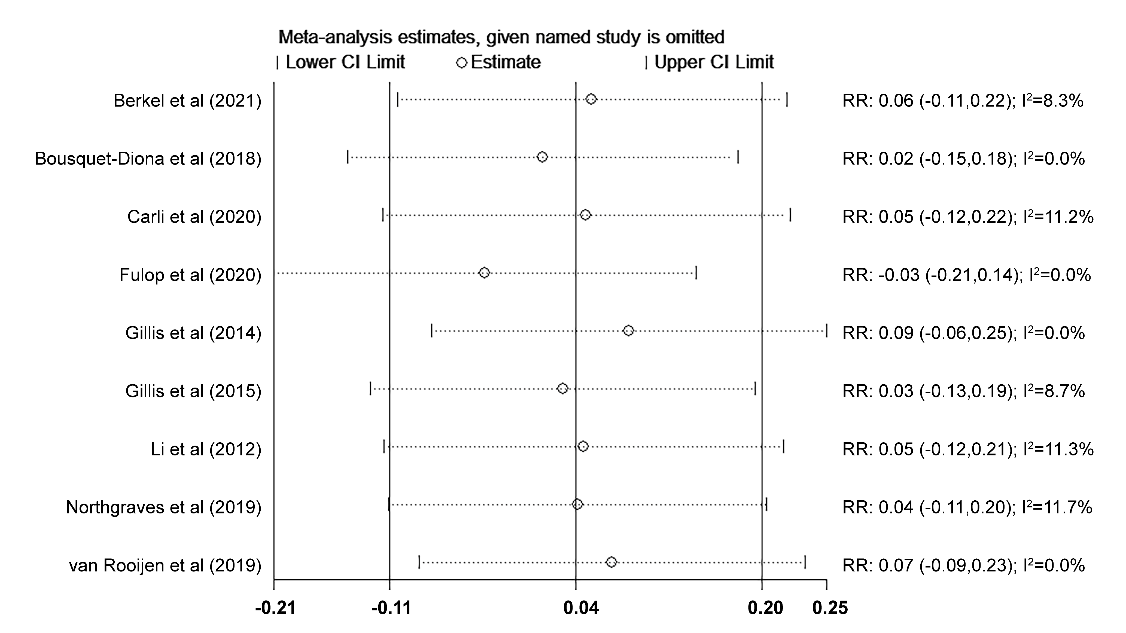


**Supplemental Fig S9.** Sensitivity analysis for LOS
